# Supplementary material for: Experiences of women in prenatal, childbirth, and postpartum care during the COVID-19 pandemic in selected cities in Brazil: The resignification of the experience of pregnancy and giving birth
Source: PLoS One. 2023 May 5;18(5):e0284773. doi: 10.1371/journal.pone.0284773 (PMC10162534; doi:10.1371/journal.pone.0284773)
Supplement: Attachment — Submitted filename: Plos One.pdf [file pone.0284773.s001.pdf]

// Plos One

de Plos One Plos One

Data de envio: 30-nov-2022 01:47PM (UTC-0200)
Identificação do Envio: 1967303975
Nome do arquivo: PONE-D-22-19394_reviewer.pdf (951.63K)
Contagem de palavras: 12184
Contagem de caracteres: 65399

PLOS ONE

Experiences of women in prenatal, childbirth and postpartum health care during the COVID-19 pandemic in selected cities in Brazil: the resignification of the experience of pregnancy and giving birth

--Manuscript Draft--

**Manuscript Number:**	PONE-D-22-19394
**Article Type:**	Research Article
**Full Title:**	Experiences of women in prenatal, childbirth and postpartum health care during the COVID-19 pandemic in selected cities in Brazil: the resignification of the experience of pregnancy and giving birth
**Short Title:**	Experiences of women in prenatal, childbirth and postpartum health care in the COVID-19 pandemic in Brazil
**Corresponding Author:**	Erika Barbara Abreu Fonseca Thomaz<br>Federal University of Maranhao: Universidade Federal do Maranhao<br>Sao Luis, MA BRAZIL
**Keywords:**	Pandemics; Prenatal Care; Parturition; Postpartum; Women's Health Services.
**Abstract:**	The COVID-19 pandemic has impacted public and private health systems around the world, impairing good practices in women's health care. However, little is known about the experiences, knowledge and feelings of Brazilian women in this period. The objective was to analyze the experiences of women, assisted in maternity hospitals accredited to the Brazilian Unified Health System (SUS), regarding health care during pregnancy, childbirth and the postpartum period, their interpersonal relationships, and perceptions and feelings related to the pandemic. This was a qualitative, exploratory research, carried out in three Brazilian municipalities with women hospitalized in 2020 during pregnancy, childbirth or postpartum period, with COVID-19 or not. For data collection, we used semi-structured individual interviews (in person, by telephone or digital platform), which have been recorded and transcribed. The content analysis in the thematic modality was displayed by the following axes: i) Knowledge about the disease; ii) Search for health care in prenatal, childbirth and postpartum period; iii) Experience with the disease; iv) Income and work; and v) Family dynamics and social support network. A total of 46 women were interviewed in São Luís-MA, Pelotas-RS and Niterói-RJ. The communication media use was important in disseminating information and fighting fake news. The pandemic negatively impacted access to health care in prenatal, childbirth and the postpartum period, contributing to the worsening of the population's social and economic vulnerabilities. Women experienced very different manifestations of the disease, but psychic disorders were very frequent. Social isolation in the pandemic disrupted the support network of these women, who found social support strategies in communication technologies. Women-centered care – including qualified listening and mental health support – can reduce the severity of COVID-19 cases in pregnant, parturient and postpartum women. Sustainable employment and income maintenance policies are essential to mitigate social vulnerabilities and reduce risks for these women.
**Order of Authors:**	Zeni Carvalho Lamy
	Erika Barbara Abreu Fonseca Thomaz
	Aluísio Gomes da Silva-Junior
	Gisele Caldas Alexandre
	Maria Teresa Seabra Soares de Britto e Alves
	Ruth Helena de Souza Britto Ferreira de Carvalho
	Letícia Oliveira de Menezes
	Sandro Schreiber de Oliveira
	Maurício Moraes

Powered by Editorial Manager® and ProduXion Manager® from Aries Systems Corporation

	Yasmim Bezerra Magalhães
	Tatiana Raquel Selbmann Coimbra
	Betzabe Butron Riveros
	Lely Stella Guzman-Barrera
**Additional Information:**	
**Question**	**Response**
**Financial Disclosure**<br><br>Enter a financial disclosure statement that describes the sources of funding for the work included in this submission. Review the submission guidelines for detailed requirements. View published research articles from *PLOS ONE* for specific examples.<br><br>This statement is required for submission and **will appear in the published article** if the submission is accepted. Please make sure it is accurate.<br><br>**Unfunded studies**<br>Enter: *The author(s) received no specific funding for this work.*<br><br>**Funded studies**<br>Enter a statement with the following details:<br>• Initials of the authors who received each award<br>• Grant numbers awarded to each author<br>• The full name of each funder<br>• URL of each funder website<br>• Did the sponsors or funders play any role in the study design, data collection and analysis, decision to publish, or preparation of the manuscript?<br>• **NO** - Include this sentence at the end of your statement: *The funders had no role in study design, data collection and analysis, decision to publish, or preparation of the manuscript.*<br>• **YES** - Specify the role(s) played.<br><br>* typeset	The authors are grateful for the technical and financial support of the Bill and Melinda Gates Foundation [INV-017424], World Health Organization (WHO) and Pan American Health Organization (PAHO) [CON20-00012173] - ZCL, EBAFT, AGSJ, GCA, MTSSBA, RHSBFC, LOM, SSO, MM, YBM, TRSC, BBR, LSGB. Also, the National Council for Scientific and Technological Development (Conselho Nacional de Desenvolvimento Científico e Tecnológico – CNPq acronym in Portuguese) [processes 306592/2018-5 (EBAFT), 314939/2020-2 (ZCL), 311479/2020-2 (MRSSBA) and 308917/2021-9 (EBAFT)] and the Coordination for the Improvement of Higher Education Personnel (Coordenação de Aperfeiçoamento de Pessoal de Nível Superior – CAPES acronym in Portuguese) [finance code 001] (EBAFT, MTSSBA, RHSBFC, ZCL) for support for scientific publication.
**Competing Interests**<br><br>Use the instructions below to enter a competing interest statement for this submission. On behalf of all authors, disclose any competing interests that	The authors have declared that no competing interests exist.

could be perceived to bias this work—acknowledging all financial support and any other relevant financial or non-financial competing interests.<br><br>This statement is required for submission and **will appear in the published article** if the submission is accepted. Please make sure it is accurate and that any funding sources listed in your Funding Information later in the submission form are also declared in your Financial Disclosure statement.<br><br>View published research articles from *PLOS ONE* for specific examples.<br><br>**NO authors have competing interests**<br><br>Enter: *The authors have declared that no competing interests exist.*<br><br>**Authors with competing interests**<br><br>Enter competing interest details beginning with this statement:<br><br>*I have read the journal's policy and the authors of this manuscript have the following competing interests: [insert competing interests here]*<br><br>* typeset	
**Ethics Statement**<br><br>Enter an ethics statement for this submission. This statement is required if the study involved:<br><br>• Human participants<br>• Human specimens or tissue<br>• Vertebrate animals or cephalopods<br>• Vertebrate embryos or tissues<br>• Field research<br><br>Write "N/A" if the submission does not require an ethics statement.	The research was approved by the Research Ethics Committee of the University Hospital of the Federal University of Maranhão, CAAE number 35645120.9.0000.5086, in compliance with resolution 466/12 of the National Health Council and by the Research Ethics Committee of the Pan American Health Organization, PAHOERC Ref. No. 0260.02. The anonymity of the interviewees was preserved. The names were replaced by codes that started with the first letter of the municipality name (N – Niterói; P – Pelotas; and S – São Luís), followed by the serial number of the interview, the age of the woman interviewed and to which period the recorded text referred to (pregnancy, childbirth or postpartum period). When the interviewee was referring to the care received in the health care network, the place of care (MRH - usual risk maternity; MAR - high risk maternity; and UBS - basic health care facility, acronyms in Portuguese) was inserted at the end of the coding.

Powered by Editorial Manager® and ProduXion Manager® from Aries Systems Corporation

3 General guidance is provided below. Consult the submission guidelines for detailed instructions. **Make sure that all information entered here is included in the Methods section of the manuscript.**<br><br>**Format for specific study types**<br><br>**Human Subject Research (involving human participants and/or tissue)**<br>• Give the name of the institutional review board or ethics committee that approved the study<br>• Include the approval number and/or a statement indicating approval of this research<br>• Indicate the form of consent obtained (written/oral) or the reason that consent was not obtained (e.g. the data were analyzed anonymously)<br><br>**Animal Research (involving vertebrate animals, embryos or tissues)**<br>• Provide the name of the Institutional Animal Care and Use Committee (IACUC) or other relevant ethics board that reviewed the study protocol, and indicate whether they approved this research or granted a formal waiver of ethical approval<br>• Include an approval number if one was obtained<br>• If the study involved *non-human primates*, add *additional details* about animal welfare and steps taken to ameliorate suffering<br>• If anesthesia, euthanasia, or any kind of animal sacrifice is part of the study, include briefly which substances and/or methods were applied<br><br>**Field Research**<br><br>Include the following details if this study involves the collection of plant, animal, or other materials from a natural setting:<br>• Field permit number<br>• Name of the institution or relevant body that granted permission	
**Data Availability**<br><br>Authors are required to make all data	No - some restrictions will apply

Powered by Editorial Manager® and ProduXion Manager® from Aries Systems Corporation

underlying the findings described fully available, without restriction, and from the time of publication. PLOS allows rare exceptions to address legal and ethical concerns. See the PLOS Data Policy and FAQ for detailed information.<br><br>A Data Availability Statement describing where the data can be found is required at submission. Your answers to this question constitute the Data Availability Statement and **will be published in the article**, if accepted.<br><br>**Important:** Stating 'data available on request from the author' is not sufficient. If your data are only available upon request, select 'No' for the first question and explain your exceptional situation in the text box.<br><br>Do the authors confirm that all data underlying the findings described in their manuscript are fully available without restriction?	
**[illegible]scribe where the data may be found in [illegible] sentences. If you are copying our sample text, replace any instances of XXX with the appropriate details.**<br><br>• If the data are **held or will be held in a public repository**, include URLs, accession numbers or DOIs. If this information will only be available after acceptance, indicate this by ticking the box below. For example: *All XXX files are available from the XXX database (accession number(s) XXX, XXX.)*.<br>• If the data are all contained **within the manuscript and/or Supporting Information files**, enter the following: *All relevant data are within the manuscript and its Supporting Information files.*<br>• If neither of these applies but you are able to provide **details of access elsewhere**, with or without limitations, please do so. For example:<br><br>*Data cannot be shared publicly because of [XXX]. Data are available from the*	Because this is a qualitative research, with in-depth interviews, addressing sensitive issues from the point of view of identifying women, and, considering that the statements that support our findings and conclusions were made available in the manuscript, we believe that the availability of the entire transcription of the focus groups can violate the ethical precepts of guaranteeing the secrecy and privacy of the participants. So, we did not make available all the data (transcription of all speeches), but we have inserted several excerpts from the speeches of the women (de-identified) throughout the manuscript.

Powered by Editorial Manager® and ProduXion Manager® from Aries Systems Corporation

XXX Institutional Data Access / Ethics Committee (contact via XXX) for researchers who meet the criteria for access to confidential data.<br><br>*The data underlying the results presented in the study are available from (include the name of the third party and contact information or URL).*<br>• This text is appropriate if the data are owned by a third party and authors do not have permission to share the data.<br><br>* typeset	
Additional data availability information:	

Powered by Editorial Manager® and ProduXion Manager® from Aries Systems Corporation

Manuscript Click here to access/download;Manuscript;2_Manuscript_Women.docx

Experiences of women in prenatal, childbirth and postpartum health care during the COVID-19 pandemic in selected cities in Brazil: the resignification of the experience of pregnancy and giving birth

Experiences of women in prenatal, childbirth and postpartum health care in the COVID-19 pandemic in Brazil

Zeni Carvalho Lamy[1], Erika Barbara Abreu Fonseca Thomaz[2], Aluísio Gomes da Silva-Junior[3], Gisele Caldas Alexandre[4], Maria Teresa Seabra Soares de Britto e Alves[5], Ruth Helena de Souza Britto Ferreira de Carvalho[6], Letícia Oliveira de Menezes[7], Sandro Schreiber de Oliveira[8], Maurício Moraes[9], Yasmim Bezerra Magalhães[10], Tatiana Raquel Selbmann Coimbra[11], Betzabe Butron Riveros[12], Lely Stella Guzman-Barrera[13]

[1]Zeni Carvalho Lamy. Public Health Department. Federal University of Maranhão. São Luís, MA, Brazil. Email: zeni.lamy@ufma.br ORCID: https://orcid.org/0000-0002-9332-0542

[2]Erika Barbara Abreu Fonseca Thomaz. Public Health Department. Federal University of Maranhão. São Luís, MA, Brazil. Email: erika.barbara@ufma.br ORCID: https://orcid.org/0000-0003-4156-4067

[3]Aluísio Gomes da Silva-Junior. Fluminense Federal University. Niterói, RJ, Brazil. Email: agsilvajunior@id.uff.br ORCID: https://orcid.org/0000-0003-2445-3963

[4]Gisele Caldas Alexandre. Fluminense Federal University. Niterói, RJ, Brazil. Email: giselealexandre@id.uff.br ORCID: https://orcid.org/0000-0002-1720-0960

[5]Maria Teresa Seabra Soares de Britto e Alves. Public Health Department. Federal University of Maranhão. São Luís, MA, Brazil. Email: mtssb.alves@ufma.br ORCID: https://orcid.org/0000-0002-4806-7752

[6]Ruth Helena de Souza Britto Ferreira de Carvalho. Public Health Department. Federal University of Maranhão. São Luís, MA, Brazil. Email: ruth.britto@ufma.br ORCID: https://orcid.org/0000-0003-1180-1586

[7]Letícia Oliveira de Menezes. Universidade Católica de Pelotas, Pelotas, RS, Brazil. Email: menezes_leticia@yahoo.com.br ORCID: https://orcid.org/0000-0001-5393-8770

[8]Sandro Schreiber de Oliveira. Universidade Federal do Rio Grande, Pelotas, RS, Brazil. Email: prof.sandro.med@gmail.com ORCID: https://orcid.org/0000-0003-4150-4515

[9]Maurício Moraes. Universidade Federal do Rio Grande, Pelotas, RS, Brazil. Email: mumana74@gmail.com ORCID: https://orcid.org/0000-0002-6538-088X

[10]Yasmim Bezerra Magalhães. Centro Universitário do Planalto Central Apparecido dos Santos, Uniceplac. Brasília, DF, Brazil. Email: yasmimbma@gmail.com ORCID: https://orcid.org/0000-0002-0614-5644

[11]Tatiana Raquel Selbmann Coimbra. PAHO/WHO. Brasília, DF, Brazil. Email selbmantat@gmail.com ORCID: https://orcid.org/0000-0001-9337-3081

[12]Betzabe Butron Riveros. PAHO/WHO. Washington, D.C. United States of America. Email: butronbe@paho.org ORCID: https://orcid.org/0000-0003-0712-0972

[13]Lely Stella Guzman-Barrera. PAHO/WHO. Brasília, DF, Brazil. Email: guzmanlel@paho.org ORCID: https://orcid.org/0000-0003-2074-312X

Correspondence author

Erika Barbara Abreu Fonseca Thomaz. Public Health Department. Federal University of Maranhão. São Luís, MA, Brazil. Email: erika.barbara@ufma.br

Abstract

The COVID-19 pandemic has impacted public and private health systems around the world, impairing good practices in women's health care. However, little is known about the experiences, knowledge and feelings of Brazilian women in this period. The objective was to analyze the experiences of women, assisted in maternity hospitals accredited to the Brazilian Unified Health System (SUS), regarding health care during pregnancy, childbirth and the postpartum period, their interpersonal relationships, and perceptions and feelings related to the pandemic. This was a qualitative, exploratory research, carried out in three Brazilian municipalities with women hospitalized in 2020 during pregnancy, childbirth or postpartum period, with COVID-19 or not. For data collection, we used semi-structured individual interviews (in person, by telephone or digital platform), which have been recorded and transcribed. The content analysis in the thematic modality was displayed by the following axes: i) Knowledge about the disease; ii) Search for health care in prenatal, childbirth and postpartum period; iii) Experience with the disease; iv) Income and work; and v) Family dynamics and social support network. A total of 46 women were interviewed in São Luís-MA, Pelotas-RS and Niterói-RJ. The communication media use was important in disseminating information and fighting fake news. The pandemic negatively impacted access to health care in prenatal, childbirth and the postpartum period, contributing to the worsening of the population's social and economic vulnerabilities. Women experienced very different manifestations of the disease, but psychic disorders were very frequent. Social isolation in the pandemic disrupted the support network of these women, who found social support strategies in communication technologies. Women-centered care – including qualified listening and mental health support – can reduce the severity of COVID-19 cases in pregnant, parturient and postpartum women. Sustainable employment and income maintenance policies are essential to mitigate social vulnerabilities and reduce risks for these women.

Keywords: Pandemics; Prenatal Care; Parturition; Postpartum; Women's Health Services.

Introduction

The COVID-19 pandemic has impacted public and private health systems around the world [1-4]. Faced with the large and unexpected demand for medium and high complexity services, the health care network and clinical protocols needed to be reorganized to meet the growing number of cases of the disease, contain the high mortality rates, and maintain the provision of essential services, such as prenatal care, delivery, and childbirth [5-12].

The World Health Organization (WHO) / Pan American Health Organization (PAHO) initiative "*COVID-19: Mitigating indirect effects on essential health services for neonates, children, adolescents and the elderly*" [13], funded by the Bill Gates Foundation, supported 20 priority countries in the world. For the Region of the Americas, Bolivia and Brazil were selected. The initiative evaluated (and encouraged) the maintenance of essential services as a result of the unfolding of the COVID-19 pandemic, identifying how the pandemic has affected the achievement of the goals defined in the Sustainable Development Goals. Therefore, it aimed to identify and contextualize actions in providing essential health services for vulnerable populations, and it identified the experiences in health care of women during pregnancy, childbirth and postpartum period and of children as one of the priority areas.

In Brazil, evidence points to changes in care for women and children [14-15], with potential damage to good care practices, since prenatal care [16]. At first, with all the efforts aimed at fighting the pandemic, there were bottlenecks providing outpatient and hospital services considered elective, as well as lack of an organized and structured network of primary services to ensure the provision of health care. In this context, it has been necessary to consider how Brazilian health services were organized for maintaining healthcare in response to situations of greater vulnerability, given the worsening of access and quality problems that already existed before the pandemic [17], including access barriers to mechanical ventilation and intensive care for obstetric patients [18]. Three of them have been identified as key aspects

responsible for maternal deaths in Brazil [19]. First, delay in identifying pregnant women's COVID-19-related symptoms and testing them. Second, delay in hospitalization of women after COVID-19 diagnosis. Health services were reluctant to admit pregnant women with severe acute respiratory symptoms, claiming that maternity hospitals were allegedly not prepared to admit patients with COVID-19. Third, delay in providing intensive care after hospitalization, such as admission to the intensive care unit (ICU), invasive ventilation, and early induction of labor. So, appropriate management of pregnancy during the COVID-19 pandemic is a challenging issue [20].

The challenge for the Brazilian Unified Health System (SUS) was how to effectively respond to a disease that, despite not having high lethality, could cause the collapse of the health system. The geographic characteristics, the population size and the Brazilian regional peculiarities brought additional difficulties for the measures to tackle the problem, which should be carried out according to the specificities of each location. Another challenge is the context of a health system with budgetary problems and already overloaded. The search for an effective strategic planning for coping must consider the guiding principles of the SUS and the impact scenario not only in the dimension of morbidity and mortality, but essentially in the context and dynamics of people's lives and their social and economic consequences.

Therefore, given the problems related to health care provision to women [14-16, 18, 20], the increased risk of dying [21, 22], the psychological suffering brought about by the pandemic [23-25] and the uncertainties experienced in this period [5], several studies have investigated the experiences, knowledge and feelings of these women during pregnancy, childbirth and postpartum period [23, 25-34]. However, we did not identify other qualitative studies, using triangulation of methods and data collection techniques, with a multicentric approach, that had interviewed women with different levels of obstetric risk, living in Brazilian municipalities with different sociodemographic characteristics, with and without a history of infection by COVID-

19 during pregnancy or hospitalization for childbirth. Therefore, the objectives of this study were to analyze the experiences of women regarding the health care offered to pregnant, parturient and postpartum women, their interpersonal and family relationships and the perceptions of risk and feelings related to the environment of coping with the pandemic, in three Brazilian municipalities. The study raised key issues for maternal and child care and provided subsidies for the planning, monitoring and evaluation of physical and mental health policies aimed at mitigating the problems and advancing the quality of care, and for coordination with income and employment maintenance policies.

Methods

Research type, period and place

This was a qualitative, exploratory research, carried out from November 2020 to May 2021, in SUS-accredited maternity hospitals in the municipalities of São Luís - Maranhão (MA), Pelotas - Rio Grande do Sul (RS) and Niterói - Rio de Janeiro (RJ). The study is part of the PAHO/WHO project entitled "*Identification of the indirect effects of COVID-19 on essential services for pregnant women, neonates, children, adolescents and the elderly at the subnational level in Brazil*", as part of the WHO initiative "*COVID-19: Mitigating indirect effects on essential health services for neonates, children, adolescents and the elderly*" [13].

In São Luís, with an estimated population of 1,115,932 inhabitants in 2021 [35], the study included two high-complexity maternity hospitals, referral centers for COVID-19 patients, which provided isolation area for pregnant and postpartum women with suspicion or confirmation of COVID-19, as well as a Maternal and Neonatal Intensive Care Unit (ICU).

In Pelotas, with an estimated population of 343,826 inhabitants in 2021 [35], the study included two maternity hospitals that serve patients through the SUS in the municipality, but only one of them was been used as a referral center for patients with COVID-19. However, patients who had COVID-19 at some point during pregnancy, childbirth or the postpartum period, regardless of the maternity hospital where they were hospitalized, were included.

In Niterói, with an estimated population of 516,981 inhabitants [35], a usual risk maternity hospital was included, a referral center for childbirth in the municipality. Although it was not the referral maternity hospital for the care of women with COVID-19, isolated spaces were created for the management of symptomatic patients when necessary. During the study, there were no hospitalizations of women with COVID-19.

The diversity in the characteristics of the maternity hospitals aimed at expanding the universe of the study and analyzing different experiences lived by women.

Study participants and sampling

The participants were women assisted in the maternity hospitals of the study. Both women hospitalized for childbirth and those who had been hospitalized for treatment of COVID-19, during pregnancy, childbirth and postpartum period, from March to November 2020, were included.

The sample was intentionally defined considering the sociodemographic and clinical characteristics of the women, seeking to include different phases of the pregnancy-puerperal cycle, age, clinical status, marital status, number of children, education, income and place of residence, in addition to the diagnosis of COVID-19. Women were interviewed in the 3 municipalities, 17 in São Luís, 12 in Pelotas and 17 in Niterói. To close the sample, the meaning saturation technique was used, which indicates the interruption of the collection when further data do not bring new information to the object studied [36].

Study techniques and tools

In the three cities of the research, an individual semi-structured interview was used, based on a previously prepared script with questions about the experiences of pregnancy, childbirth and birth during the COVID-19 pandemic. Before the interviews, a structured questionnaire was filled out with sociodemographic, cultural and clinical history data of each woman and newborn child.

The interviews were conducted face-to-face, via telephone or digital platform, according to the interviewees' choice, on the most convenient days and time for them. When in person, all the necessary safety measures (use of mask, distancing, hand washing and use of gel alcohol) were followed. The average duration was 50 minutes and the interviews were recorded and later transcribed. Each municipality had a team of interviewers trained for the role.

Data collection

The first step was to contact the research sites to identify the participants. This took place in the context of the development of the technical product "*Identification of strategies, experiences and evidence-based initiatives, which was already underway in the selected region, in Brazil*", to mitigate the indirect effects of the pandemic on five vulnerable population groups (pregnant women, newborns, children, adolescents and the elderly), as part of the response to the pandemic according to the demographic, geographic, epidemiological and care context. In our research, a sub-project was created for women. A list of all women admitted to the maternity hospitals until the data collection start date was made available. Based on this list, a review of medical records was conducted seeking patient characteristics data to define the sample. These data were organized in a table for each maternity hospital and categorized based on the characteristics found, constructing typologies, in which women were separated into groups.

Then, telephone contact was initiated to present the research and invitation to participate. There was no direct refusal. However, after three unsuccessful contact attempts, the participant was replaced by another from the same group of identity characteristics.

Analysis

Content analysis was performed in the thematic modality as proposed by Bardin, 2011 [37], and Minayo, 2014 [38]. The results were presented through thematic axes, organized from the synthesis of the interviews.

Ethical considerations

The research was approved by the Research Ethics Committee of the University Hospital of the Federal University of Maranhão, CAAE number 35645120.9.0000.5086, in compliance with resolution 466/12 of the National Health Council and by the Research Ethics Committee of the Pan American Health Organization, PAHOERC Ref. No. 0260.02. The anonymity of the interviewees was preserved. The names were replaced by codes that started with the first letter of the municipality name (N – Niterói; P – Pelotas; and S – São Luís), followed by the serial number of the interview, the age of the woman interviewed and to which period the recorded text referred to (pregnancy, childbirth or postpartum period). When the interviewee was referring to the care received in the health care network, the place of care (MRH - usual risk maternity; MAR - high risk maternity; and UBS - basic health care facility, acronyms in Portuguese) was inserted at the end of the coding.

Results and discussion

Sociodemographic characteristics of women

We interviewed 46 women, from 17 to 40 years old, in the three municipalities, and the largest age group was of women from 24 to 34 years old. The majority declared to be black or mixed race (in Pelotas, white), married or in a consensual union (living with a partner and children), declared to be evangelical or Catholic, and mainly residing in urban areas (in São Luís, three lived in rural areas). Most had completed high school, except in Pelotas, where a higher proportion of women had higher education. Most said they were employed (including self-employed), with a monthly family income of up to three minimum wages and receiving emergency financial aid (in São Luís, incomes of up to one minimum wage predominated, and in Pelotas there was a slight predominance of incomes of up to three minimum wages).

Most women were primiparous or had two children (in São Luís, one pregnant woman was multiparous with 16 pregnancies and 11 children). All had access to prenatal care, however, with a variation in the number of consultations (from 4 to 10). Public basic facilities were the main referral centers for prenatal care in all the cities studied. In São Luís, some pregnant women, given the risk factors, were also followed up in outpatient clinics of the maternity hospitals. In Pelotas and Niterói, there was a greater number of visits to private establishments for prenatal care and complementary exams.

A normal development of pregnancy was described by most of the women, without complications and comorbidities. However, in São Luís, seven women reported comorbidities. Also in São Luis, there was a higher proportion of cesarean deliveries and premature births (one stillbirth). Most deliveries in Pelotas and Niterói were vaginal and at term (in Pelotas, seven of the interviewees had not yet given birth at the time of the interview).

Thematic axes that emerged from the interviewees' statements

The results are displayed by five thematic axes: i) Knowledge about the disease; ii) Search for health care in prenatal, childbirth and postpartum period; iii) Experience of being affected

by COVID-19; iv) Income and work; and v) Family dynamics and social support network. Fragments of the interviewed women's statements are presented in Table 1 to illustrate each thematic axis.

Table 1. Fragments of the interviewees' statements. São Luís-MA, Niteroi-RJ and Pelotas-RS, Brazil. 2021.

Thematic axes	Fragments of the interviewees' statements
**Knowledge about the disease, its prevention and risk perception**	*"I was very afraid of getting COVID, not only for myself, but for my baby, so I avoided using public transportation, and remained indoors as much as possible." (N01, 26 years old, about the gestational period)*
	"No, I think our greatest feeling was that of uncertainty, you know, the feeling of not knowing what is going to happen, of fear, of anguish. Anyway, everything is OK, soon we will be locked up, and we do not know." (P01, 29 years old, about the gestational period)
	"Watching TV, I was scared because I was afraid of my children getting it, I was afraid of staying at the hospital and bringing the disease to my son, or my son getting it in the hospital, I was very afraid. Thank goodness nothing happened." (P11, 18 years old, about the gestational period)
	"Look! At first, I wasn't scared, right? Because I never imagined that I would catch it, (because it was) on the other side of the world, right?" (S19, 30 years old, about the gestational period)
	"I learned about it from the newspapers, right? And... I just knew it was out there, not in Brazil. Then it came to Brazil and so on. But it was more in the television, in the newspapers. Just that." (S04, 21 years old, about the gestational period)
**Search for health care in prenatal, childbirth and postpartum period**	*"The prenatal care, which are the family centers, I have nothing to complain about them, they are great, attentive, cautious, they always make appointments month after month, I did all the free consultations quarterly, with results on time, in all the appointments I was assisted by the wonderful doctor who has there, thank goodness." (N02, 22 years old, about the gestational period, UBS)*
	"Oh, you have to do the ultrasound", they gave me the paper and told me to go there. "Go to the clinic, go somewhere else and so on". (N03, 17 years old, about the gestational period, UBS)

	"I had to pay for the 'ultras' as it takes a long time to get the referral, so I had to pay to be able to advance the process for the doctor." (N04, 30 years old, about the gestational period, UBS)<br><br>*"He was born, then they took him to take the vaccine, cleaned him and immediately brought him to me… Then my mother took him for about 15 minutes for me to finish having the stitches, taking a shower, the nurses helped me take a shower quickly, then immediately he was breastfed, he had the first contact with me… Then I was with him until the discharge." (N05, 23 years old, about the postpartum period, MRH)*
	"Yes, consultations, some tests, ultrasounds, I had to pay for them all." (P08, 34 years old, about the gestational period)
	"It was then that I started doing prenatal care at the health care facility, because the pandemic was already there, then before I did my prenatal care as it was risky because of diabetes, they were not attending people there because of that, right? Then the nurse came and said: 'Now you are going to come here at the facility to take these vaccines and talk to us online on the cell phone,' so that was it." (S09, 33 years old, about pregnancy, UBS)<br><br>*"Yes, for sure (the pandemic affected my prenatal care). Because we have to have the follow up, and everything must be quite right. And then we couldn't go to the health facility, because there were, especially here, the facility in (name of the neighborhood) there were a lot of people. So we also didn't have that ease of moving around, going to other places, also because of… this thing that we don't know who has it, who doesn't, you know? And for, for those who are pregnant, ah, it's much easier to catch, because we have (low) immunity, right?" (S10, 42 years old, about pregnancy, UBS)*<br><br>*"Oh no, I didn't have (a companion). My mother, who went with me every time, they did not let her in. She went (and stayed) outside the maternity hospital." (S16, 17 years old, about childbirth, MAR)*
**Experience of being affected by COVID-19**	*"I didn't look for anything and I didn't even go to the health center, because, as my husband had it and he had to be isolated and stay here, as we are poor, there's no way to isolate ourselves, the tendency in our home was for everyone to catch it, but God was so good that only me, my oldest son and his father had it. The little ones didn't catch it." (N06, 31 years old, about the postpartum period)*
	"Look, I felt a lot of weakness. I was no longer able to move. I was quite… just throwing up. I felt short of breath. I felt all the symptoms, all of them!" (S12, 24 years old, about the period of ICU stay during pregnancy, MAR)

	"When did I return? From COVID… I waited… I decided to do one more isolation at home. So, as there was a spare room in my mother-in-law's house and I wasn't sure if she had it […]. So I said like this: 'No. I'm going to stay in the isolated room,' And I stayed for about ten days in the isolation that I decided to do myself." (S1, 28 years old, about the gestational period)<br><br>*"Yes, I could breastfeed her, because it didn't imply anything… COVID with breast milk." (S13, 28 years old, about the postpartum period)*
**Income and work**	*"I used to work with nails, right, there in downtown Niterói and because of the pandemic, isolation, people could not see each other, and it affected me financially." (N02, 22 years old, about the gestational period)*<br><br>*"I was at home, because as soon as I got pregnant, that's when the pandemic started. I had to be in isolation. I worked there for 2 weeks and then it closed. I work in day care and then it had to close." (N06, 31 years old, about the gestational period)*
	"But my mother has a store and she needed to go back to open the store, right? Because that's where she makes her living, we pay the bills by her sales, and… I had to go to my boyfriend's house. And that's when I ended up getting it. Because he also needed to buy construction stuff and he had to go out." (S01, 28 years old, gestational period).
**Family dynamics and social support network**	*"Regarding the pandemic, it was very difficult, very difficult, due to the isolation, the distance from my family and my mother. I only came here with my husband, I'm young, right, my first pregnancy that went ahead, so it was very difficult, I felt distressed, very sad, and especially impotent, I couldn't do anything to solve it." (N02, 22 years old, about the gestational period)*
	"Yeah, I had more support from my husband, my daughter, that they were in the house with me, that whole thing, and from friends over the phone." (P09, 34 years old about the gestational period)<br><br>*"Look, I've always been a party girl, actually. I've always liked people a lot, agglomerations, everything that can't be done now. In this case, but in the current situation, I thought about doing it closer to the birth and doing a "tea-drive" in which people just pass by and take a souvenir and with a mask, with all the precautions, but not in person, right, even because there is no way." (P03, 27 years old, about the gestational period)*
	"So, it changed on my husband's part, who changed a lot. We kept our distance because of the disease" (S05, 40 years old, about the gestational period)

	"At the time, [he] couldn't be there. Yeah…my family too. To this day, there are people in my family who still don't know her [daughter]. Because… to prevent many people from coming here. Although we already go out, but there are people who haven't had it yet, we don't know if they still have the symptoms. So a lot of people still don't know her, and she's already grown up. On the part of her father's family, many people still don't know her. Her grandmother, from her father side, still doesn't know her." (S03, 32 years old, about the postpartum period, MAR)<br><br>*"No. The other [son, the eldest] only came after… he came when he was two months old, he came to meet his little brother. He stayed with grandma. From his father's side." (S06, 29 years old, about the postpartum period)*<br><br>*"… only with my husband that I could count on. Only with him. The people who were helping me, like my family, everything is far, far away. Then who I have to count on are my children here. The people from the church and God, right?" (S11, 40 years old, about the postpartum period)*

MRH: habitual risk maternity (*maternidade de risco habitual* – acronym in Portuguese). MAR: high risk maternity (*maternidade de alto risco* – acronym in Portuguese). UBS: Basic Health Care Facility (*Unidade Básica de Saúde* – acronym in Portuguese).

Knowledge about the disease, its prevention and risk perception

Initially, the seriousness of the problem and news on deaths seemed far away to the women. News on the severity of the pandemic came from abroad and its progression in Brazil was perceived with less impact. The information that pregnant women had access to COVID-19 news only through television and social media confirms the idea that the disease was not yet part of their reality.

The proximity to the real risk of a little-known virus with unpredictable effects on the health of the population was realized when the pandemic was no longer only in the media and reached the cities of residence of these women, that is, it began to affect well-known characters and to be witnessed in their own lives, and in the lives of relatives and close friends. Associated with this, local authorities began to impose measures to prevent the spread of the disease, raising

emotions and attitudes related to the health condition, the adequacy of new routines, changes in the forms of access to the health care network (RAS) and to goods and services.

Despite the information provided mentioning pregnant women as a population at risk for infection by COVID-19, and the need for care, some of the women, in São Luís, believed they would not become infected and maintained their lives without further restrictions. Others, on the other hand, began to take preventive measures due to the occurrence of the disease in someone close to them.

COVID-19 infection and even measures to contain the pandemic – such as social isolation, physical distancing and quarantine – have affected the mental health of women in several countries [25, 39]. Studies have shown an increase in the prevalence of minor psychological disorders during pregnancy and postpartum period [23, 24]. High levels of stress, anxiety, and/or depression [40, 41] in addition to sadness, worries, fear, anger, irritation, frustration, loneliness, guilt, doubt, and conflict about receiving health care [34, 41] may have been a reflection of the uncertainties and difficulties experienced by these women in the experience of gestating and giving birth in a pandemic caused by a new and unknown disease [5, 42]. In Brazil, the large volume of information about the disease and the dissemination of contradictory or false news, in a short period of time, also contributed to the insecurity of these women [43].

Search for health care in prenatal, childbirth, postpartum period and childcare

When the pandemic started, access to prenatal care was difficult in all the cities studied. At that time, COVID-19 vaccination had not yet begun. The closing of facilities for routine care, the redirection of pregnant women to prenatal care in other health facilities, the prioritization of care for respiratory symptoms, the illness and/or removal of professionals due

to risk factors for COVID-19 and the fear of commuting by public transportation led to a discontinuity in prenatal care.

With the objective of alleviating the inconvenience generated with the changes in the RAS, some health facilities in São Luís and Pelotas used communication technology, telemedicine, for prenatal follow-up and maintenance of care. Communication technologies can offer new possibilities to maintain links between professionals and patients, monitor pregnant women, especially those affected by COVID-19, avoiding discontinuity of care [44, 45]. However, the implementation strategy of these telemedicine devices must overcome the difficulties of acceptance and trust on the part of patients and ensure alternation with face-to-face consultations.

A considerable number of women, mainly in Niterói and Pelotas, sought to complement their consultations and exams in the private network. However, it was reported in Pelotas that, even in the private network, there was discontinuity of care or replacement by remote service, through communication applications and telephone calls. Some of the women mentioned the lack of trust in the new modalities of remote assistance, and those who adhered better mentioned their previous bond with the physician.

A few mentioned receiving follow-up by other professionals such as nurses. In Niterói, some of the pregnant women reported visits by Community Health Agents as a differential approach in pregnancy monitoring. Other professionals, such as nurses, can participate in supporting pregnant and postpartum women in terms of guidance and monitoring (in person or remotely) of pregnancy and COVID-19, increasing the bond with the team and providing reassurance to them [41, 46].

The greatest difficulty reported in all the cities studied was related to ultrasound exams, laboratory tests and vaccination, which, when available, were conducted in centralized facilities, far from the women's area of residence. This required travel in a private car or in

private vehicles, considered safer alternatives to public transportation in terms of agglomeration and infection risk, but generated unforeseen costs in their economy.

RAS must be kept open for pregnant and postpartum women, ensuring prenatal care in Primary Care, facilitating access to the necessary tests, ensuring the continuity and safety of care and reinforcing humanization measures in the relationship with women, such as qualified listening, reception, bonding and accountability.

Considering the regional inequalities in the provision of health services already pointed out by Viacava et al., 2018 [47] and Albuquerque et al. [48, 49], in which the North and Northeast regions have lower access patterns than the South and Southeast regions, access barriers in four dimensions suggested by Oliveira et al., 2019 [50], were identified in the studied municipalities: i) no geographic access, since there were no health care facilities in some neighborhoods and/or localities; ii) availability of facilities, professionals and exams in a timely number and response time; iii) acceptability of replacing professionals in prenatal care, in safety procedures against COVID-19 adopted by professionals and in remote forms of care (introduction of telemedicine devices); and iv) feasibility (payment capacity), considering the requirement of spending on private transportation and payment for consultations and exams in the private sector.

Access to a maternity facility in childbirth was a relevant topic in the women's statements. This was the longest and most detailed item in the reports, in a clear demonstration of the importance attributed to this aspect. This perception stands out, especially in São Luís, given the characteristics of the women interviewed (affected by COVID-19 and with comorbidities). Barriers to admission to childbirth were identified since entering the service, even before the first consultation with the health professional, until the moment of childbirth itself. A common point that emerged from the pregnant women's statements was the long waiting time during labor, indicating a delay in meeting their needs. At the time of delivery, the women's statements

showed flaws in the acceptability of care, in the way it was provided. Infection protection measures, reorganization of health services and overburdened professionals were seen by women as obstacles to access. A feeling of "dehumanization of the professionals" was perceived when the professionals did not show support for the user's complaints or when the service did not offer the technological resources that were expected and understood as necessary for the adequate monitoring of labor. These access barriers may also be understood in terms of availability, due to the small number of maternity hospitals with exclusive beds for patients with COVID-19. Added to this fact is the insufficient resources available to pay for travel to the maternity hospital, considering the long distance to be traveled to obtain care.

In Pelotas and Niterói, access seems to have been easier. Pregnant women reported good reception and care during childbirth. They highlighted the sensitivity of the team in relaxing some social distance rules to privilege humanization aspects such as 92 the presence of a (single) companion during labor. This fact was reported prominently by them. In Niterói, the redirection 101 of pregnant women not affected by COVID-19 by the basic health care facilities to the studied maternity hospital attracted pregnant women from all neighborhoods of the city. Even when there was a state maternity hospital closer to their homes, in some cases, pregnant women preferred to be referred to the maternity facility under study. The fact that the State Maternity Hospital also received symptomatic and affected pregnant women led those without symptoms, fearing infection, to seek the Municipal Maternity Hospital.

In São Luís, perhaps due to greater severity 85 of COVID-19 in pregnant women, compared to the other two municipalities, the presence of a companion in the maternity hospitals was not allowed, and this was a source of complaint from the women. They also reported that some professionals, more sensitive to the situation of isolation of women, provided more attention to their demands and even used their own phones as a means of communication between families and the parturients. The concern with aspects of the humanization of care for pregnant women

was highlighted by Estrela et al., 2020, [51] as fundamental in mitigating women's fears and insecurities, as well as promoting well-being and preventing complications in their mental health [5, 34, 41, 42], as provided in the technical notes of the Ministry of Health regarding care for pregnant women with COVID-19 [52, 53].

The COVID-19 pandemic also impacted access to puerperal consultations and routine vaccinations, generating a certain discontinuity in this care in all the municipalities studied. Some of the women interviewed, especially in São Luís and Niterói, mentioned problems in scheduling appointments at maternity clinics and basic health care facilities, such as lack of referral, unavailability of agenda and late scheduling. Chisini et al., 2021 [16], observed a reduction in the provision of consultations throughout Brazil during the year 2020.

Reduced provision of antenatal consultations, health infrastructure overloading and potentially harmful policies implemented with little evidence have also been reported in other countries [39]. Although the impacts of the pandemic have been greater in low- and middle-income countries [3, 54], even in developed countries, pregnant and postpartum women have experienced interruptions in access to obstetric health care. The perceived quality of obstetric care was negatively influenced by the interruption of services, cancellation of face-to-face consultations, replacement by virtual and/or telephone consultations, and exclusion of the companion during hospitalization for childbirth [27]. In addition to problems in the provision of health services, pregnant women and mothers with newborns may experience difficulties in accessing services due to interruptions in transportation, lockdown measures or reluctance to come to health care facilities due to fear of infection [55].

Experience of being affected by COVID-19

In the cities studied, the experience of illness among women was different. In São Luís, 14 were hospitalized, five of them in the ICU. In Niterói, the only pregnant woman who

reported having had COVID-19 during pregnancy had mild symptoms. In Pelotas, most women had mild symptoms such as tiredness, loss of smell, and runny nose. Only two women experienced shortness of breath, but this did not result in hospitalization. Pregnant women and mothers are not at greater risk of COVID-19 infection than those who are not pregnant. However, symptomatic women may experience more adverse outcomes compared to non-pregnant women and appear to disproportionately face socioeconomic consequences [39].

All the women interviewed expressed the perception of the inability to avoid infection as one of their frustrations, as they said they complied with safety recommendations, such as social isolation of family members, restrictive measures for home visits, use of a mask, alcohol-based hand rub, bathing and changing clothes when arriving from external environments and being careful to avoid sharing utensils. Some women mentioned that they had expectations about the moment of delivery that were altered by circumstances. Shuman et al., 2022 [56], commented on the frustration of North American women in the idealized experiences of pregnancy and childbirth that were very different from those experienced during the pandemic. In contexts of greater social vulnerability, pregnant and postpartum women feel helpless, and the joyful event of pregnancy turns into fear and stress [41].

Many Brazilian women were infected during pregnancy, childbirth and postpartum period, with a high impact on maternal morbidity and mortality. In this sense, the pandemic may impact the achievement of the Sustainable Development Goals. Before the pandemic, Brazil was expected to achieve a 46.6% reduction in the maternal mortality ratio by 2030 [57]. However, Brazil had the highest rates of maternal mortality due to COVID-19, worldwide [21, 22]. Limited resources for the provision of health care [54], the lack of women-centered care [19], the inefficiency of health systems and the inability to adequately manage the pandemic [58] contributed to the worsening of the adverse impacts of the pandemic in reproductive health; and emphasized the need for appropriate measures for adequate antenatal and postnatal care.

Income and work

The work status of pregnant women was greatly affected by the pandemic in all municipalities. The preventive removal of those who had contact with the public in trade and service activities, the low customer demand and the removal due to illness of them and/or their spouses, significantly modified the dynamics of work and income in the families. The vast majority of women sought emergency aid (Federal) as a way to supplement the income lost because of the pandemic. In Niterói, the municipal government developed a broad income guarantee policy [59] including emergency aid with municipal resources.

There were complaints from women affected by COVID-19, who resorted to Social Security disease aid, about delay in granting or not receiving the benefit. The income situation is sensitive as a social determinant for health problems, especially COVID-19 [60]. In other countries, women were also more vulnerable than men to losing their income due to the pandemic; and working mothers struggled with increased childcare demands [39]. International bodies recommend the development of employment and income maintenance policies in the pandemic, as observed in some countries [61]. When resuming comprehensive care, it is necessary to consider the income of women, especially the most vulnerable, pregnant women and those with children in early childhood.

Family dynamics and social support network

As to the social support network, most women reported having had the direct presence of a partner or family member during pregnancy, childbirth and postpartum period. Despite reporting this social contact, they mentioned a routine that made significant use of communication technologies (phone calls, video calls and messages through communication applications), responsible for creating a support different from what was expected for this stage

of life, but recognized as important and essential during the course of pregnancy and the birth of babies. The distance from family and friends, despite being seen as necessary, was a gap identified by the pregnant women that left them helpless from the daily help they had as an expectation.

The maintenance of social support networks plays an important role in daily life and in household and work tasks, as well as in the well-being of women, linking family and friends according to availability and safety conditions. Social support is defined as a buffer against stressors and it has emotional, informational and instrumental aspects, and is also a protective factor against postpartum depression. Communication technologies and social media can play an important role when social isolation is established [60].

Final considerations

The experience of the pandemic by women was different in the three municipalities. The pandemic had a negative impact on access to prenatal care, humanized childbirth, puerperal consultations and the routine vaccination service, areas of primary health care that require strengthening planning in the three spheres of health management.

The availability of information about the disease and its prevention provided more tranquility to women and greater adherence to care flows and procedures indicated by the teams. The media played a very important role in the dissemination of information and in combatting fake news or information without scientific evidence. It is necessary to invest in reliable sources of information, based on the best scientific evidence, delivered in a timely manner, to better guide professionals and the population.

The stress naturally generated in pregnancy has been greatly amplified by the changes brought about by the pandemic. Careful listening by professionals and mental health support can reduce stress and prevent more serious conditions such as depression.

The pandemic contributed to the worsening of the population's social and economic vulnerability. Policies for maintaining employment and income are essential for mitigating this vulnerability and improving the population's adherence to prevention and social isolation measures.

Although most of the women interviewed had some type of family support during the period, social isolation was responsible for disrupting the social support network. The maintenance of these networks plays an important role in daily life and in household and work tasks, as well as in the well-being of women, linking family and friends according to availability and safety conditions. Communication technologies played an important role in the reorganization of the social support network.

Acknowledgments

The authors are grateful for the technical and financial support of the Bill and Melinda Gates Foundation [INV-017424], World Health Organization (WHO) and Pan American Health Organization (PAHO) [CON20-00012173]. Also, the National Council for Scientific and Technological Development (Conselho Nacional de Desenvolvimento Científico e Tecnológico – CNPq acronym in Portuguese) [processes 306592/2018-5, 314939/2020-2, 311479/2020-2, and 308917/2021-9] and the Coordination for the Improvement of Higher Education Personnel (Coordenação de Aperfeiçoamento de Pessoal de Nível Superior – CAPES acronym in Portuguese) [finance code 001] for support for scientific publication. We would also like to thank the Federal University of Maranhão, the Fluminense Federal University, the Municipal Health Foundation of Niteroi, the Federal University of Rio Grande, the Catholic University of Pelotas, the Municipal Health Departments of São Luís, Niterói e Pelotas, the State Health Department of Maranhão, Rio de Janeiro e Rio Grande do Sul, and to the women who participated in this research.

References

1. Chen Y, Li Z, Zhang YY, Zhao WH, Yu ZY. Maternal health care management during the outbreak of coronavirus disease 2019. J Med Virol. 2020;92: 731-739. doi: 10.1002/jmv.25787.
2. Lumbreras- Marquez MI, Campos- Zamora M, Leon HLD, Farber MK. Maternal mortality from COVID- 19 in Mexico. Int J Gynecol Obstet. 2020;150: 266–267. doi: 10.1002/ijgo.13250.
3. McDonald CR, Weckman AM, Wright JK, Conroy AL, Kain KC. Pregnant women in low- and middle-income countries require a special focus during the COVID-19 pandemic. Front Glob Womens Health. 2020;1: 564560. doi: 10.3389/fgwh.2020.564560.
4. Ng QX, Lee EZ, Tay JA, Arulanandam S. Impact of COVID-19 'circuit-breaker' measures on emergency medical services utilisation and out-of-hospital cardiac arrest outcomes in Singapore. Emerg Med Australas. 2021;33: 149-151. doi: 10.1111/1742-6723.13668.
5. Du L, Gu Y, Cui M, Li W, Wang J, Zhu L, et al. Investigation on demands for antenatal care services among 2 002 pregnant women during the epidemic of COVID-19 in Shanghai. Zhonghua Fu Chan Ke Za Zhi. 2020;55: 160-165. doi: 10.3760/cma.j.cn112141-20200218-00112.
6. Esegbona-Adeigbe S. Impact of COVID-19 on antenatal care provision. Eur J Midwifery. 2020;4: 16. doi: 10.18332/ejm/121096.
7. Hong Z, Li N, Li D, Li J, Li B, Xiong W, et al. Telemedicine during the COVID-19 pandemic: experiences from Western China. J. Med. Internet Res. 2020;22: e19577. doi: 10.2196/19577.
8. Jain K, Bhatia N, Grewal A, Pandya ST, Gupta S, Bagga R, et al. Management of pregnant laboring women during COVID-19 pandemic. J Anaesthesiol Clin Pharmacol. 2020;36: S91-S96. doi: 10.4103/joacp.JOACP_258_20.

9. Wu H, Sun W, Huang X, Yu S, Wang H, Bi X,et al. Online antenatal care during the COVID-19 pandemic: opportunities and challenges. J. Med. Internet Res. 2020;22: e19916. doi: 10.2196/19916.

10. Derya YA, Altiparmak S, Akça E, Gökbulut N, Yilmaz AN. Pregnancy and birth planning during COVID-19: The effects of tele-education offered to pregnant women on prenatal distress and pregnancy-related anxiety. Midwifery. 2021;92: 102877. doi: 10.1016/j.midw.2020.102877.

11. Dongarwar D, Ajewole VB, Harris K, Oduguwa E, Ofili TU, Onyenaka C, et al. A framework for protecting pregnant women in the era of COVID-19 pandemic. Int J MCH AIDS. 2021;10: 109-112. doi: 10.21106/ijma.419.

12. Jakubowski D, Sys D, Kajdy A, Lewandowska R, Kwiatkowska E, Cymbaluk-Płoska A, et al. Application of telehealth in prenatal care during the COVID-19 pandemic—A cross-sectional survey of Polish women. J Clin Med. 2021;10: 2570. doi: 10.3390/jcm10122570.

13. World Health Organization. Maintaining the provision and use of services for maternal, newborn, child and adolescent health and older people during the COVID-19 pandemic: Lessons learned from 19 countries. Washington: WHO; 2021. [cited 2022 June 17]. Available from: https://apps.who.int/iris/bitstream/handle/10665/351108/9789240040595-eng.pdf?sequence=1&isAllowed=y

14. Costa ML, Souza RT, Pacagnella RC, Bento SF, Ribeiro-do-Valle CC, Luz AG, et al. Facing the COVID-19 pandemic inside maternities in Brazil: A mixed-method study within the REBRACO initiative. PLoSONE. 2021;16: e0254977. doi: 10.1371/journal.pone.0254977.

15. Stofel NS, Christinelli D, Silva RCS, Salim NR, Beleza ACS, Bussadori JCC. Perinatal care in the COVID-19 pandemic: analysis of Brazilian guidelines and protocols. Revista

Brasileira de Saúde Materno Infantil. 2021;21: 89-98. doi: 10.1590/1806-9304202100s100005.

16. Chisini LA, Castilhos ED, Costa FS, D'Avila OP. Impact of the COVID-19 pandemic on prenatal, diabetes and medical appointments in the Brazilian National Health System. Revista Brasileira de Epidemiologia. 2021;24: e210013. doi: 10.1590/1980-549720210013.

17. Bittencourt SDA, Domingues RMSM, Reis LGC, Ramos MM, Leal MC. Adequacy of public maternal care services in Brazil. Reprod Health. 2016;13:120. doi: 10.1186/s12978-016-0229-6

18. Takemoto MLS, Menezes MO, Adreucci CB, Nakamura-Pereira M, Amorim MMR, Katz L, et al. The tragedy of COVID-19 in Brazil: 124 maternal deaths and counting. Int J Gynaecol Obstet. 2020;151: 154-156. doi: 10.1002/ijgo.13300.

19. Diniz D, Brito L, Rondon G. Maternal mortality and the lack of women-centered care in Brazil during COVID-19: Preliminary findings of a qualitative study. The Lancet Regional Health Americas. 2022; 10: 100239. doi: 10.1016/j.lana.2022.100239.

20. Sahin D, Tanacan A, Erol SA, Yetiskin FDY, Besimoglu B, Tokalioglu EO, et al. Management of pregnant women with COVID- 19: A tertiary pandemic center experience on 1416 cases. J Med Virol. 2021;94:1074-1084. doi: 10.1002/jmv.27423.

21. PAHO. PAHO Director urges countries to prioritize pregnant and lactating women for COVID-19 vaccinations. 2021 [cited 2022 June 16]. Available from: https://www.paho.org/en/news/8-9-2021-paho-director-urgescountries-prioritize-pregnant-and-lactating-women-covid-19.

22. Souza ASR, Amorim RAM. Maternal mortality by Covid-19 in Brazil. Rev Bras Saude Mater Infant. 2021; 21: S253-S256. doi: 10.1590/1806-9304202100S100014.

23. Keating NE, Dempsey B, Corcoran S, McAuliffe FM, Lalor J, Higgins MF. Women's experience of pregnancy and birth during the COVID-19 pandemic: a qualitative study.Ir J Med Sci. 2021: 1-8. doi: 10.1007/s11845-021-02862-2.

24. Matvienko-Sikar K, Pope J, Cremin A, Carr H, Leitao S, Olander EK, Meaney S. Differences in levels of stress, social support, health behaviours, and stress-reduction strategies for women pregnant before and during the COVID-19 pandemic, and based on phases of pandemic restrictions, in Ireland. Women Birth. 2021;34: 447-454. doi: 10.1016/j.wombi.2020.10.010.

25. Talbot J, Charron V, Konkle ATM. Feeling the void: Lack of support for isolation and sleep difficulties in pregnant women during the COVID-19 pandemic revealed by twitter data analysis. Int J Environ Res Public Health. 2021;18: 393. doi: 10.3390/ijerph18020393.

26. Jaffe E, Sullivan K, Spach N, Munson A, Goldfarb I. "Coronavirus has changed everything": Experiences of pregnancy and birth among women impacted by the COVID-19 pandemic. Am J Obstet Gynecol. 2020;223: 979. doi: 10.1016/j.ajog.2020.08.157.

27. Brislane A, Larkin F, Jones H, Davenport MH. Access to and quality of healthcare for pregnant and postpartum women during the COVID-19 pandemic. Front Glob Womens Health. 2021;2: 628625. doi: 10.3389/fgwh.2021.628625.

28. Izhar R, Husain S, Tahir MA, Husain S, Hussain S. Pregnant during the COVID-19 pandemic: Knowledge, concerns, attitudes and practices of Pakistani women. Eur J Midwifery. 2021;5: 54. doi: 10.18332/ejm/142818.

29. Kaur TP, Rana A, Perumal V, Sharma A, Dadhwal V, Kulshrestha V, et al. A cross-sectional analysis to evaluate knowledge, attitude and practices among pregnant women during COVID-19 pandemic. J Obstet Gynaecol India. 2021;71: 1-10. doi: 10.1007/s13224-021-01558-y.

30. Marbán-Castro E, Pons-Duran C, García-Otero L, Chen H, Herrera LB, Gil MDM, et al. Acceptability of clinical trials on COVID-19 during pregnancy among pregnant women and healthcare providers: A qualitative study. Int J Environ Res Public Health. 2021;18: 10717. doi: 10.3390/ijerph182010717.

31. Onchonga D, Alfatafta H, Ngetich E, Makunda W. Health-seeking behaviour among pregnant women during the COVID-19 pandemic: A qualitative study. Heliyon. 2021;7: e07972. doi: 10.1016/j.heliyon.2021.e07972.

32. Rezaei F, Masaeli Z, Atighechian G. Information needs of pregnant women in the COVID-19 pandemic from experts' point of view: A qualitative study. Int J Community Based Nurs Midwifery. 2021;9: 139-151. doi: 10.30476/IJCBNM.2021.87447.1432.

33. Atmuri K, Sarkar M, Obudu E, Kumara A. Perspectives of pregnant women during the COVID-19 pandemic: A qualitative study. Women Birth. 2022;35: 280-288. doi: 10.1016/j.wombi.2021.03.008.

34. Mirzakhani K, Shoorab NJ, Akbari A, Khadivzadeh T. High-risk pregnant women's experiences of the receiving prenatal care in COVID-19 pandemic: a qualitative study. BMC Pregnancy Childbirth. 2022;22: 363. doi: 10.1186/s12884-022-04676-1.

35. Instituto Brasileiro de Geografia e Estatística – IBGE. Cidades e Estados. 2021 [cited 2022 June 17]. Available from: https://www.ibge.gov.br/cidades-e-estados

36. Minayo MCS. Amostragem e saturação em pesquisa qualitativa: consensos e controvérsias. Revista Pesquisa Qualitativa. 2017;5:1-12. [cited 2022 June 17]. Available from: https://editora.sepq.org.br/rpq/article/view/82/59

37. Bardin L. Análise de Conteúdo. 1st ed. Lisboa: Edições 70; 2011.

38. Minayo MCS. O desafio do conhecimento: pesquisa qualitativa em saúde. 14 ed. São Paulo: Hucitec; 2014.

39. Kotlar B, Gerson E, Petrillo S, Langer A, Tiemeier H. The impact of the COVID-19 pandemic on maternal and perinatal health: a scoping review. Reprod Health. 2021;18: 10. doi:10.1186/s12978-021-01070-6

40. Stampini V, Monzani A, Caristia S, Ferrante G, Gerbino M, Pedrini A, et al. The perception of Italian pregnant women and new mothers about their psychological wellbeing, lifestyle, delivery, and neonatal management experience during the COVID-19 pandemic lockdown: a web-based survey. BMC Pregnancy Childbirth. 2021;21: 473. doi: 10.1186/s12884-021-03904-4.

41. Akhter S, Kumkum FA, Bashar F, Rahman A. Exploring the lived experiences of pregnant women and community health care providers during the pandemic of COVID-19 in Bangladesh through a phenomenological analysis. BMC Pregnancy Childbirth. 2021;21: 810. doi: 10.1186/s12884-021-04284-5.

42. Fakari FR, Simbar M. Coronavirus pandemic and worries during pregnancy; a letter to editor. Arch Acad Emerg Med. 2020;8: e21. PMID: 32185371.

43. Galhardi CP, Freire NP, Minayo MCS, Fagundes MCM. Fact or Fake? An analysis of disinformation regarding the Covid-19 pandemic in Brazil. Cien Saude Colet. 2020;25: 4201-4210. doi: 10.1590/1413-812320202510.2.28922020.

44. Bokolo AJ. Exploring the adoption of telemedicine and virtual software for care of outpatients during and after COVID-19 pandemic. Ir J Med Sci. 2021;190:1-10. doi: 10.1007/s11845-020-02299-z.

45. Almuslim H, Aldossary S. Models of incorporating telehealth into obstetric care during the COVID-19 pandemic, its benefits and barriers: A scoping review. Telemedicine and e-Health. 2022;28: 24-38. doi: 10.1089/tmj.2020.0553.

46. Sweet L. COVID-19 Special Issue – The Impact of COVID-19 on women, babies, midwives, and midwifery care. Women and Birth. 2022;35: 211-212. doi: 10.1016/j.wombi.2022.03.002.

47. Viacava F, Oliveira RAD, Carvalho CC, Laguardia J, Bellido JG. SUS: oferta, acesso e utilização de serviços de saúde nos últimos 30 anos. Ciência & Saúde Coletiva. 2018;23: 1751-1762. doi: 10.1590/1413-81232018236.06022018.

48. Albuquerque MV, d'Ávila-Viana AL, Lima LD, Ferreira MP, Fusaro ER, Iozzi FL. Desigualdades regionais na saúde: mudanças observadas no Brasil de 2000 a 2016. Ciência & Saúde Coletiva. 2017;22: 1055-1064. doi: 10.1590/1413-81232017224.26862016.

49. Albuquerque MVR, Leandro LH. Desigualdade, situação geográfica e sentidos da ação na pandemia da COVID-19 no Brasil. Cadernos de Saúde Pública. 2020;36: e00208720. doi: 10.1590/0102-311X00208720.

50. Oliveira PAD, Duarte CMR, Pavão ALB, Viacava F. Barriers in access to services in five Health Regions of Brazil: perceptions of policymakers and professionals in the Brazilian Unified National Health System. Cadernos de Saúde Pública. 2019; 35: e00120718. doi: 10.1590/0102-311X00120718.

51. Estrela F M, Silva KKA, Cruz MA, Gomes NP. Gestantes no contexto da pandemia da Covid-19: reflexões e desafios. Physis: Revista de Saúde Coletiva. 2020;30: e300215. doi: 10.1590/S0103-73312020300215.

52. Brasil. Ministério da Saúde. Secretaria de Atenção Primária à Saúde. Departamento de Ações Programáticas Estratégicas. Coordenação-Geral de Ciclos da Vida. Coordenação de Saúde das Mulheres. Nota Técnica Nº7/2020-COSMU/CGCIVI/DAPES/SAPS/MS. Atenção às Gestantes no Contexto da Infecção COVID-19 Causada pelo Novo Coronavírus (SARS-CoV-2). 2020 [cited 2022 April 22]. Available from: https://www.sogesp.com.br/media/2161/nota-tecnica-7-ms_gestantes-covid-19.pdf.

53. Brasil. Ministério da Saúde. Secretaria de Atenção Primária à Saúde. Departamento de Ações Programáticas Estratégicas. Coordenação-Geral de Ciclos da Vida. Coordenação de Saúde das Mulheres. Nota Técnica Nº6/2020-COSMU/CGCIVI/DAPES/SAPS/MS. Atenção às Gestantes no Contexto da Infecção SARS-CoV-2. 2020 [cited 2022 May 22]. Available from: http://www.enf.ufmg.br/images/SEI_MS_-_0014128689_-_Nota_Tecnica.pdf.pdf.

54. Amorim MMR, Takemoto MLS, Fonseca EB. Maternal deaths with coronavirus disease 2019: a different outcome from low- to middle-resource countries? Am J Obstet Gynecol. 2020;223: 298-299. doi: 10.1016/j.ajog.2020.04.023.

55. UNICEF. Maternal and newborn health and COVID-19. UNICEF Data. 2020. [cited 2022 April 20]. Available from: https://data.unicef.org/topic/maternal-health/covid-19/.

56. Shuman CJ, Morgan ME, Chiangong J, Pareddy N, Veliz P, Peahl AF, et al. "Mourning the experience of what should have been": Experiences of peripartum women during the COVID-19 pandemic. Matern Child Health J. 2022;26: 102-109. doi:10.1007/s10995-021-03344-8.

57. Motta CT, Moreira MR. Will Brazil comply with the SDG 3.1 of the 2030 Agenda? An analysis of maternal mortality, from 1996 to 2018. Ciência & Saúde Coletiva. 2021;26: 4397-4409. doi: 10.1590/1413-812320212610.10752021.

58. Chmielewska B, Barratt I, Townsend R, Kalafat E, Meulen JVD, Gurol-Urganci I, et al. Effects of the COVID19 pandemic on maternal and perinatal outcomes: a systematic review and meta-analysis. Lancet Glob Health. 2021;9: e759-e772. doi: 10.1016/S2214-109X(21)00079-6.

59. Silva-Junior AG, Latgé PK, Oliveira RAT, Franco CM, Vasconcelos MCV. A experiência de Niterói no enfrentamento da COVID 19: notas preliminares sobre a articulação de políticas sociais e de saúde. APS em Revista. 2020;2: 128-136. doi: 10.14295/aps.v2i2.126.

60. Guimarães RM, Araújo Eleutério T, Costa Monteiro-da-Silva JH. Estratificação de risco para predição de disseminação e gravidade da COVID-19 no Brasil. Rev Bras Estud Popul. 2020;37: 1-17. doi: 10.20947/S0102-3098a0122.

61. Baptista I, Marlier E, Spasova S, Peña-Casas R, Fronteddu B, Ghailani D, et al. Social protection and inclusion policy responses to the COVID-19 crisis. An analysis of policies in 35 countries. European Social Policy Network (ESPN). Luxembourg: Publications Office of the European Union; 2021. [cited 2022 April 25]. Available from: https://ec.europa.eu/social/main.jsp?langId=en&catId=89&newsId=10065&furtherNews=yes#navItem-1.

62. Alhomaizi A, Alhomaizi D, Willis S, Verdeli H. Social distancing in the era of COVID-19: A call for maintaining social support for the maternal population. Glob Health Sci Pract. 2021;9: 229-237. doi: 10.9745/GHSP-D-20-00398.

712

Plos One

RELATÓRIO DE ORIGINALIDADE

36%	34%	33%	23%
ÍNDICE DE SEMELHANÇA	FONTES DA INTERNET	PUBLICAÇÕES	DOCUMENTOS DOS ALUNOS

FONTES PRIMÁRIAS

1	Submitted to Mississippi State University<br>Documento do Aluno	5%
2	www.wjgnet.com<br>Fonte da Internet	4%
3	Submitted to Universiti Kebangsaan Malaysia<br>Documento do Aluno	3%
4	www.frontiersin.org<br>Fonte da Internet	3%
5	www.scielo.br<br>Fonte da Internet	2%
6	hopkinshumanitarianhealth.org<br>Fonte da Internet	1%
7	journals.plos.org<br>Fonte da Internet	1%
8	wjgnet.com<br>Fonte da Internet	1%
9	ijier.net<br>Fonte da Internet	<1%

10 Debora Diniz, Luciana Brito, Gabriela Rondon. "Maternal mortality and the lack of women-centered care in Brazil during COVID-19: Preliminary findings of a qualitative study", The Lancet Regional Health - Americas, 2022 <1 %
Publicação

11 assets.researchsquare.com <1 %
Fonte da Internet

12 www.preprints.org <1 %
Fonte da Internet

13 ouci.dntb.gov.ua <1 %
Fonte da Internet

14 www.thieme-connect.com <1 %
Fonte da Internet

15 journals.lww.com <1 %
Fonte da Internet

16 midirs.org <1 %
Fonte da Internet

17 www.jbra.com.br <1 %
Fonte da Internet

18 bmcpregnancychildbirth.biomedcentral.com <1 %
Fonte da Internet

19 Katharine G Norris, Phoebe A Huang, J. Christopher Glantz, Ruth-Sally Kodam, Martina Anto-Ocrah. "A Cross-Cultural <1 %

Analysis of the COVID-19 Pandemic's Impact on Antenatal Healthcare-Seeking Behaviors in Ghana and the United States", Journal of Patient Experience, 2021
Publicação

20	fjfsdata01prod.blob.core.windows.net<br>Fonte da Internet	<1 %
21	www.researchsquare.com<br>Fonte da Internet	<1 %
22	Submitted to Flinders University<br>Documento do Aluno	<1 %
23	ijmm.ir<br>Fonte da Internet	<1 %
24	Eynas Omer Ibrahim Abdalla, Seema Nahid, Sikha Shastham Valappil, Srinivas Gudavalli et al. "Impact of COVID-19 status on patients receiving neuraxial analgesia during labor: A national retrospective-controlled study", Qatar Medical Journal, 2022<br>Publicação	<1 %
25	revistas.unal.edu.co<br>Fonte da Internet	<1 %
26	scielo.iec.gov.br<br>Fonte da Internet	<1 %
27	www.medrxiv.org<br>Fonte da Internet	<1 %

28	doi.org<br>Fonte da Internet	<1 %
29	Steven Ndugwa Kabwama, Rhoda K. Wanyenze, Suzanne N. Kiwanuka, Alice Namale et al. "Interventions for Maintenance of Essential Health Service Delivery during the COVID-19 Response in Uganda, between March 2020 and April 2021", International Journal of Environmental Research and Public Health, 2022<br>Publicação	<1 %
30	Clara V. I. Ponto, Glady I. Rambert, Mayer F. Wowor. "Description of SGOT and SGPT Levels in Pregnant Women with Confirmed COVID-19", Medical Scope Journal, 2022<br>Publicação	<1 %
31	teses.usp.br<br>Fonte da Internet	<1 %
32	Everton Daniel Rauber, Gabriele Rissotto Menegazzo, Jessica Klöckner Knorst, Gabriela Bohrer Bolsson, Thiago Machado Ardenghi. "Pathways between toothache and children' oral health - related quality of life", International Journal of Paediatric Dentistry, 2020<br>Publicação	<1 %
33	hdl.handle.net<br>Fonte da Internet	

<1 %

34	ripe-tomato.org<br>Fonte da Internet	<1 %
35	Submitted to Curtin University of Technology<br>Documento do Aluno	<1 %
36	David Natali. "COVID-19 and the opportunity to change the neoliberal agenda: evidence from socio-employment policy responses across Europe", Transfer: European Review of Labour and Research, 2022<br>Publicação	<1 %
37	Hayman Win, Sohana Shafique, Nicole Probst-Hensch, Günther Fink. "Change in nutritional status of urban slum children before and after the first COVID-19 wave in Bangladesh: A repeated cross-sectional assessment", PLOS Global Public Health, 2022<br>Publicação	<1 %
38	Victoria Riley, Naomi Ellis, Lorna Mackay, Jennifer Taylor. "The impact of COVID-19 restrictions on women's pregnancy and postpartum experience in England: A qualitative exploration", Midwifery, 2021<br>Publicação	<1 %
39	bmjopen.bmj.com<br>Fonte da Internet	<1 %

40	Submitted to University of Nottingham<br>Documento do Aluno	<1 %
41	Matheus Negri Boschiero, Camila Vantini Capasso Palamim, Fernando Augusto Lima Marson. "The hindrances to perform the COVID-19 vaccination in Brazil", Human Vaccines & Immunotherapeutics, 2021<br>Publicação	<1 %
42	bmchealthservres.biomedcentral.com<br>Fonte da Internet	<1 %
43	www.alliedacademies.org<br>Fonte da Internet	<1 %
44	J. Arturo Cardona-Pérez, Isabel Villegas-Mota, A. Cecilia Helguera-Repetto, Sandra Acevedo-Gallegos et al. "Prevalence, clinical features, and outcomes of SARS-CoV-2 infection in pregnant women with or without mild/moderate symptoms: Results from universal screening in a tertiary care center in Mexico City, Mexico", PLOS ONE, 2021<br>Publicação	<1 %
45	Francisco Ortega, Dominique Béhague. "Contested leadership and the governance of COVID-19 in Brazil: The role of local government and community activism", Global Public Health, 2022<br>Publicação	<1 %

46	cathedral.ojs.galoa.com.br<br>Fonte da Internet	<1 %
47	link.springer.com<br>Fonte da Internet	<1 %
48	onlinelibrary.wiley.com<br>Fonte da Internet	<1 %
49	research.one.surgery<br>Fonte da Internet	<1 %
50	Carolina Matteussi Lino, Carla Fabiana Tenani, Marília Jesus Batista. "COVID-19 em Jundiaí/SP: dinâmica temporal das notificações e análise espacial da prevalência soroepidemiológica", Revista de Epidemiologia e Controle de Infecção, 2021<br>Publicação	<1 %
51	Submitted to University of Hertfordshire<br>Documento do Aluno	<1 %
52	periodicos.ufsm.br<br>Fonte da Internet	<1 %
53	Mariana Konder, Gisele O'Dwyer. "Regulation of access to hospital beds in emergency care and the development of integrated health services", Research Square, 2019<br>Publicação	<1 %
54	Submitted to University of Sunderland<br>Documento do Aluno	<1 %

55	llufb.llu.lv<br>Fonte da Internet	<1 %
56	Anagaw Derseh Mebratie, Adiam Nega, Anna Gage, Damen Haile Mariam, Munir Kassa Eshetu, Catherine Arsenault. "Effect of the COVID-19 pandemic on health service utilization across regions of Ethiopia: An interrupted time series analysis of health information system data from 2019–2020", PLOS Global Public Health, 2022<br>Publicação	<1 %
57	Submitted to University of College Cork<br>Documento do Aluno	<1 %
58	journalijdr.com<br>Fonte da Internet	<1 %
59	publichealth.jmir.org<br>Fonte da Internet	<1 %
60	Elizabeth Mollard, Kevin Kupzyk, Tiffany Moore. "Postpartum stress and protective factors in women who gave birth in the United States during the COVID-19 pandemic", Women's Health, 2021<br>Publicação	<1 %
61	nur.tanta.edu.eg<br>Fonte da Internet	<1 %
62	psychiatryai.com	

Fonte da Internet

<1 %

63 scielosp.org
Fonte da Internet
<1 %

64 www.rbgg.com.br
Fonte da Internet
<1 %

65 Ribeiro, Marizélia Rodrigues Costa, Maria Teresa Seabra Soares de Britto e. Alves, Rosângela Fernandes Lucena Batista, Cecília Cláudia Costa Ribeiro, Lilia Blima Schraiber, Marco Antônio Barbieri, Heloisa Bettiol, and Antônio Augusto Moura da Silva. "Confirmatory Factor Analysis of the WHO Violence Against Women Instrument in Pregnant Women: Results from the BRISA Prenatal Cohort", PLoS ONE, 2014.
Publicação
<1 %

66 vip.ucaldas.edu.co
Fonte da Internet
<1 %

67 www.arca.fiocruz.br
Fonte da Internet
<1 %

68 sigaa.ufrn.br
Fonte da Internet
<1 %

69 toxilatin2018.com
Fonte da Internet
<1 %

70	Erika Barbara Abreu Fonseca Thomaz, Cláudia Maria Coelho Alves, Luciana Freitas Gomes e Silva, Cecília Cláudia Costa Ribeiro de Almeida et al. "Breastfeeding Versus Bottle Feeding on Malocclusion in Children: A Meta-Analysis Study", Journal of Human Lactation, 2018<br>Publicação	<1 %
71	dspace3.ufes.br<br>Fonte da Internet	<1 %
72	iscte-iul.pt<br>Fonte da Internet	<1 %
73	Submitted to CVC Nigeria Consortium<br>Documento do Aluno	<1 %
74	www.ncbi.nlm.nih.gov<br>Fonte da Internet	<1 %
75	Antônio Augusto Moura da Silva, Rosângela Fernandes Lucena Batista, Vanda Maria Ferreira Simões, Erika Barbara Abreu Fonseca Thomaz et al. "Changes in perinatal health in two birth cohorts (1997/1998 and 2010) in São Luís, Maranhão State, Brazil", Cadernos de Saúde Pública, 2015<br>Publicação	<1 %
76	academictree.org<br>Fonte da Internet	<1 %

77 Melanie Etti, Jackeline Alger, Sofía P. Salas, Robin Saggers et al. "Global research priorities for COVID-19 in maternal, reproductive and child health: results of an international survey", Cold Spring Harbor Laboratory, 2021 <1%
Publicação

78 repositorio.ufrn.br <1%
Fonte da Internet

79 Cristiano Alencar Arrais, Graciella Corcioli, Gabriel da Silva Medina. "The Role Played by Public Universities in Mitigating the Coronavirus Catastrophe in Brazil: Solidarity, Research and Support to Local Governments Facing the Health Crisis", Frontiers in Sociology, 2021 <1%
Publicação

80 Jenna A. LoGiudice, Susan Bartos. "Mixed-Methods Study of the Experience of Pregnancy During the COVID-19 Pandemic", Journal of Obstetric, Gynecologic & Neonatal Nursing, 2022 <1%
Publicação

81 Manuella Adrian. "Addiction and Sexually Transmitted Disease (STD), Human Immunodeficiency Virus, (HIV), and Acquired Immune Deficiency Syndrome (AIDS): Their <1%

Mutual Interactions", Substance Use & Misuse, 2009
Publicação

---

82 ebuah.uah.es
Fonte da Internet
<1 %

---

83 Priscylla de Oliveira Miranda, Ana Cassia Martins Ribeiro Cruz, Marcos Antônio Barbosa Pacheco, Rosane da Silva Dias et al. "MAIS MÉDICOS (MORE DOCTORS) PROGRAM: NARRATIVES, CHANGES AND LIMITS", Texto & Contexto - Enfermagem, 2020
Publicação
<1 %

---

84 doaj.org
Fonte da Internet
<1 %

---

85 jmrh.mums.ac.ir
Fonte da Internet
<1 %

---

86 periodicos.uepa.br
Fonte da Internet
<1 %

---

87 proceedings.science
Fonte da Internet
<1 %

---

88 rareknits.blogspot.com
Fonte da Internet
<1 %

---

89 repositorio.cruzeirodosul.edu.br
Fonte da Internet
<1 %

---

90 Aretuza Cruz Vieira, Mariana Lucas Da Rocha Cunha. "My role and responsibility: mothers'
<1 %

perspectives on overload in caring for children with cancer*", Revista da Escola de Enfermagem da USP, 2020
Publicação

91	Megan L. Gow, Chris Rossiter, Lynne Roberts, Maddison J. Henderson et al. "COVID-19, lifestyle behaviors and mental health: A mixed methods study of women 6 months following a hypertensive pregnancy", Frontiers in Public Health, 2022<br>Publicação	<1 %
92	Victor Hugo Alves Mascarenhas, Adriana Caroci-Becker, Kelly Cristina Máxima Pereira Venâncio, Nayara Girardi Baraldi et al. "Care recommendations for parturient and postpartum women and newborns during the COVID-19 pandemic: a scoping review", Revista Latino-Americana de Enfermagem, 2020<br>Publicação	<1 %
93	etui.org<br>Fonte da Internet	<1 %
94	pesquisa.bvsalud.org<br>Fonte da Internet	<1 %
95	platcovid.com<br>Fonte da Internet	<1 %
96	pubcovid19.pt<br>Fonte da Internet	

<1%

97	scholar.dgist.ac.kr<br>Fonte da Internet	<1%
98	storage.googleapis.com<br>Fonte da Internet	<1%
99	www.bjorl.org.br<br>Fonte da Internet	<1%
100	www.mdpi.com<br>Fonte da Internet	<1%
101	www.rsdjournal.org<br>Fonte da Internet	<1%
102	"International Handbook of Teaching and Learning in Health Promotion", Springer Science and Business Media LLC, 2022<br>Publicação	<1%
103	Luciana Noemí Garcia, Alejandra Destito Solján, Nicolas Falk, Nicolás Leonel Gonzalez et al. "Congenital syphilis in Argentina: experience in a pediatric hospital", Cold Spring Harbor Laboratory, 2020<br>Publicação	<1%
104	Pablo Gaitán-Rossi, Mireya Vilar-Compte, Valeria Cruz-Villalba, Nazme Sabina, Manuela Villar-Uribe. "A Qualitative Assessment of the Essential Health and Nutrition Service	<1%

Delivery in the Context of COVID-19 in Bangladesh: The Perspective of Divisional Directors", Healthcare, 2022

Publicação

105	Udita Iyengar, Bhavisha Jaiprakash, Hanako Haitsuka, Sohye Kim. "One Year Into the Pandemic: A Systematic Review of Perinatal Mental Health Outcomes During COVID-19", Frontiers in Psychiatry, 2021<br>Publicação	<1 %

Excluir citações	Desligado	Excluir correspondências	Desligado
Excluir bibliografia	Desligado		

Plos One

RELATÓRIO DE GRADEMARK

NOTA FINAL

/0

GENERAL COMMENTS

Professor

PÁGINA 1

PÁGINA 2

PÁGINA 3

PÁGINA 4

PÁGINA 5

PÁGINA 6

PÁGINA 7

PÁGINA 8

PÁGINA 9

PÁGINA 10

PÁGINA 11

PÁGINA 12

PÁGINA 13

PÁGINA 14

PÁGINA 15

PÁGINA 16

PÁGINA 17

PÁGINA 18

PÁGINA 19

PÁGINA 20

PÁGINA 21

PÁGINA 22

PÁGINA 23

PÁGINA 24

PÁGINA 25

PÁGINA 26

PÁGINA 27

PÁGINA 28

PÁGINA 29

PÁGINA 30

PÁGINA 31

PÁGINA 32

PÁGINA 33

PÁGINA 34

PÁGINA 35

PÁGINA 36

PÁGINA 37

PÁGINA 38

PÁGINA 39

PÁGINA 40

PÁGINA 41
