# Supplementary material for: Experiences of women in prenatal, childbirth, and postpartum care during the COVID-19 pandemic in selected cities in Brazil: The resignification of the experience of pregnancy and giving birth
Source: PLoS One. 2023 May 5;18(5):e0284773. doi: 10.1371/journal.pone.0284773 (PMC10162534; doi:10.1371/journal.pone.0284773)
Supplement: Attachment — Submitted filename: Response_to_Reviewers.docx [file pone.0284773.s003.docx]

PLOS ONE

PONE-D-22-19394

**Experiences of women in prenatal, childbirth, and postpartum care during the COVID-19 pandemic in selected cities in Brazil: the resignification of the experience of pregnancy and giving birth**

Dear Academic Editor and Reviewers,

Thank you for the careful evaluation of the manuscript referred to above. After careful reading, we considered all suggestions made and responded item by item in this document. We are resubmitting the manuscript to Plos One, addressing the points raised during the review process. In addition to the 'Rebuttal letter', we have also included a 'Revised Manuscript with Track Changes' and an unmarked version of the paper without tracked changes, under the instructions received.

Thank you very much for the opportunity.

Kind regards,

The Authors

**********************************************************************

Editor and Reviewer Comment:

After careful consideration, we feel that it has merit but does not fully meet PLOS ONE’s publication criteria as it currently stands. Therefore, we invite you to submit a revised version of the manuscript that addresses the points raised during the review process.

The authors have satisfactorily met all the reviewers' requests and it is almost ready for publication. However, I would request that you respond to a final (adapted) comment from one of the reviewers:

"The authors provided valid legal and ethical reasons why excerpts of speeches of all the forty-six (46) study participants would not be made publicly available without any restriction; and also provided a non-author contact information to which a data requests may be sent. However, they must include these limitations in a Data Availability Statement in the section 'Limitations of the Study'.”

**Authors' response:**

Thank you for allowing us to resubmit our manuscript to PLOS ONE. We are sending the revised version of the manuscript in response to this reviewer**'** comment. We have included this limitation in the study Discussion, as follows:

“*For ethical reasons, we could not make public the database containing the transcription of all the speeches of the women interviewed, which is a limitation for open science. However, we transcribed several excerpts of statements into the manuscript, selecting representative statements from the sample, and pointing out agreements and disagreements, when present. In addition, some de-identified data may be made available upon request.*”

We will be happy to make any further adjustments if necessary.

Thank you very much.

The authors
